# Oral Surgery Procedures in a Patient with Hajdu-Cheney Syndrome Treated with Denosumab—A Rare Case Report

**DOI:** 10.3390/ijerph18179099

**Published:** 2021-08-28

**Authors:** Magdalena Kaczoruk-Wieremczuk, Paulina Adamska, Łukasz Jan Adamski, Piotr Wychowański, Barbara Alicja Jereczek-Fossa, Anna Starzyńska

**Affiliations:** 1 Department of Oral Surgery, Medical University of Gdańsk, 7 Dębinki Street, 80-211 Gdańsk, Poland; mkaczoruk@gumed.edu.pl (M.K.-W.); paulina.adamska@gumed.edu.pl (P.A.); lukaszadamski@gumed.edu.pl (Ł.J.A.); 2 Department of Oral Surgery, Medical University of Warsaw, 6 St. Binieckiego Street, 02-097 Warsaw, Poland; piotrwychowanski@wychowanski.pl; 3 Department of Oncology and Hemato-Oncology, University of Milan, 7 Festa del Perdono Street, 20-112 Milan, Italy; barbara.jereczek@ieo.it; 4 Division of Radiotherapy, IEO European Institute of Oncology, IRCCS, 435 Ripamonti Street, 20-141 Milan, Italy

**Keywords:** tooth extraction, Hajdu-Cheney syndrome, HCS, oral surgery, genetic disorders, complicated bone healing, systemic disease

## Abstract

Background: Hajdu-Cheney syndrome (HCS) is a very rare autosomal-dominant congenital disease associated with mutations in the *NOTCH2* gene. This disorder affects the connective tissue and is characterized by severe bone resorption. Hajdu-Cheney syndrome most frequently affects the head and feet bones (acroosteolysis). Case report: We present an extremely rare case of a 34-year-old male with Hajdu-Cheney syndrome. The patient was admitted to the Department of Oral Surgery, Medical University of Gdańsk, in order to perform the extraction of three teeth. These teeth were not eligible for conservative treatment and prosthetic reconstruction. The patient was treated with denosumab (angiogenesis and receptor activator of nuclear factor-κB RANK ligand inhibitor, RANKL). Discussion: Denosumab is a monoclonal antibody against RANKL. This drug works through a suppression of osteoclast activity. In cases of patients in which the pathway of the RANK/RANKL/osteoprotegerin is dysregulated, denosumab has been approved for the treatment off-label. In patients receiving denosumab, a delayed wound healing in the oral cavity and osteonecrosis may occur. Dental procedures involving the alveolar bone process (tooth extractions and bone alveoloplasty) may be a risk factor for medication-related osteonecrosis of the jaw (MRONJ). Spontaneous osteonecrosis is rarely observed. MRONJ consists of the destruction of exposed bone, with the exposure persisting for a minimum of 6–8 weeks. This is the first article about an HCS patient treated with denosumab who underwent invasive oral surgery procedures. This case report highlights the difficulties for professionals occurring during the oral surgery procedures in such patients.

## 1. Introduction

Hajdu-Cheney syndrome (synonyms: HCS, acroosteolysis dominant type, Orpha number: 955, acroosteolysis with osteoporosis and changes in skull and mandible, arthro-dento-osteodysplasia, arthrodentoosteodysplasia, Cheney syndrome, cranioskeletal dysplasia with acroosteolysis, familial osteodysplasia, hereditary osteodysplasia with acro-osteolysis, HJCYS, and serpentine fibula-polycystic kidney syndrome/SFPKS) is a very rare autosomal-dominant disease with mutations in the *NOTCH2* gene. This disorder affects the connective tissue and is characterized by severe bone resorption. A major characteristic of this syndrome is osteolysis in head and feet bones (acroosteolysis). The most common symptoms of HCS are as follows: skull deformities, joints laxity, osteoporosis, and short stature. Sometimes, neurological disorders may occur. A normal rate of mental development is observed in the majority of patients [1,2,3,4,5,6,7,8,9,10]. To our knowledge, there have been about 50 patients reported in the literature with HCS [10]. However, in the Pubmed database, no patient with HCS who underwent oral surgery procedures was described. HCS was first described in 1948 by Hajdu and Kauntze, and then in 1965 by Cheney [1,2]. The clinical and radiological characteristics by Brennan and Pauli are described in Table 1 [10].

Hajdu-Cheney syndrome is characterized with numerous bone deformities, osteolysis, and osteoporosis. Conservative (pharmacological) and surgical treatments are used to treat the symptoms of the disease. Currently, there are no effective treatments for HCS. Treatment focuses on alleviating the effects of the disease, eliminating complications, and improving patients’ quality of life [10]. In the treatment of progressive osteoporosis, experimental drugs have been used including the following: bisphosphonates (zoledronate, pamidronate, alendronate) [4,11,12,13,14,15,16,17], denosumab [18], and glucocorticoids (e.g., prednisone) [17]. Supplementation of the primary treatment comprises the following: tri-calcium phosphate, cholecalciferol (2000 IU, daily), and subcutaneous teriparatide [17]. Bisphosphonates (BPs) are derivatives of pyrophosphate. BPs contain two phosphonate groups that connect to the centrally located carbon atom (P-C-P). They are antiresorptive and anticatabolic drugs. The P-C-P structure is responsible for the BPs affinity to hydroxyapatites, thus affecting the bone turnover [19,20,21]. Drugs included in the bisphosphonates’ group include the following: alendronate, etidronate, ibandronate, clodronate, medronate, oxydronate, pamidronate, risedronate, tiludronate, and zoledronate [22,23]. Denosumab is a monoclonal IgG2 antibody against the receptor activator of nuclear factor-kB ligand (RANKL). This drug works through a suppression of osteoclast activity. In cases of patients in whom the pathway of the RANK/RANKL/osteoprotegerin is dysregulated, denosumab has been approved for the treatment off-label, for example HCS, postmenopausal osteoporosis, male osteoporosis, or glucocorticoid-induced osteoporosis [18,24,25,26,27,28]. BPs and denosumab are drugs that can induce medication-related osteonecrosis of the jaw (MRONJ) [29]. Other substances that may cause MRONJ are as follows: monoclonal antibodies, tyrosine kinase inhibitors, radiopharmaceuticals, mammalian target of rapamycin inhibitors, selective estrogen receptor modulators, immunosuppressants, and tocilizumab (a newly classified drug related to osteonecrosis of the jaws used to treat COVID-19 patients) [30,31]. Surgical treatment of HCS patients includes orthopedic treatment, e.g., cervical kyphotic deformities [32], spinal reconstruction [33], or translation of the radius in the course of treatment of a vertebral compression fracture [34,35].

The aim of this article was to describe the difficulties for professionals occurring during the oral surgery procedures in patients with Hajdu-Cheney syndrome treated using antiresorptive or antiangiogenic drugs.

## 2. Case Presentation

In June 2016, a 34-year-old male was admitted to the Department of Oral Surgery, Medical University of Gdańsk, for the extraction of three teeth (14, 15, and 27). These teeth were not eligible for conservative treatment and prosthetic reconstruction. In the second decade of his life, the patient was diagnosed with Hajdu-Cheney syndrome. In the family history, his father was diagnosed with HCS. The patient has a confirmed heterozygous c.6206delC/p.Pro2069Glnfs*8 mutation encoded by the *NOTCH2* gene on chromosome 1 (locus 1p34).

The prenatal period and the patient’s early psychomotor development were normal. The first symptoms of HCS appeared in the second decade of his life. As a result of the lack of bone healing after fracture, the patient was diagnosed with Hajdu-Cheney syndrome. The presence of acroosteolysis was revealed, with resorption and shortening of the phalanges of the hands and feet. A significant advancement of lesions concerned the distal and middle phalanges and, to a lesser extent, the proximal phalanges. The man was diagnosed with dystrophy of epiphysis and metaphysis of long bones (metacarpus, forearms, and metatarsus) and wrist, excessive mobility of distal phalanges of the hands and feet, hyperkyphosis, hyperlordosis, scoliosis, pigeon chest, thickening of skull bones (hyperostosis), widening of lambdoid suture, skull elongation in the sagittal plane, Worm cubes, stomatognatic system abnormalities (retrogenia and microgenia), and osteoporosis. Magnetic resonance imaging of the brain and spinal cord and abdominal ultrasound showed no abnormalities. Endocrine, metabolic, and inflammatory markers were unchanged. Only the increased alkaline phosphatase level was found. The patient remains under the constant care of an orthopedic, rehabilitation, and neurological outpatients clinic. Orthopedists treat the patient using denosumab (angiogenesis and receptor activator of nuclear factor-κB RANK ligand inhibitor) and vitamin D_3_ at an oral dose of 10,000 UI, given weekly. The patient receives denosumab every six months. It has been proven that denosumab is associated with a significantly higher risk of developing MRONJ compared to zoledronic acid. The last dose of denosumab was administered 3 months before the treatment in our clinic (May 2016). The next dose of denosumab was given in November 2016. In case of denosumab, the last administration should be given at least 3 weeks before surgical procedure. The treatment can be continued after waiting at least 4–6 weeks after surgical procedures [31]. Scintigraphic examination showed a slight improvement in bone density after denosumab (first scintigraphy July 2016 vs. second scintigraphy January 2017).

**Figure 1:**
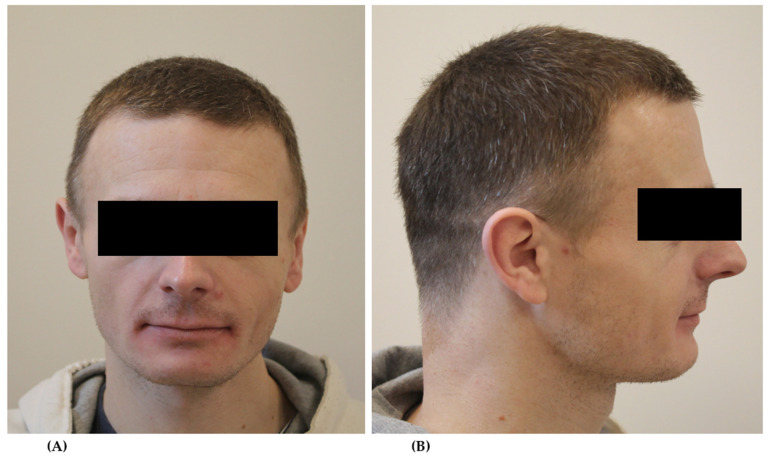
Head and face (**A**) and lateral head (**B**) photography: long philtrum, low-set ears, dolichocephaly, retrogenia, and microgenia.

**Figure 2:**
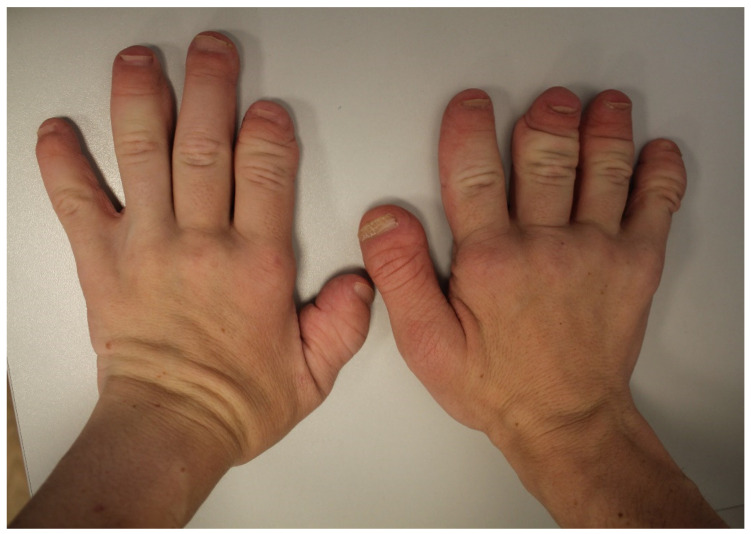
Short and thick fingers with deformations.

In the extraoral examination, the characteristic features of dysmorphism were shown, including the following: dolichocephaly, prominent eyebrows and eyelashes, eyelid gaps obliquely downward in the centrifugal direction, long philtrum, low-set ears, retrogenia and microgenia (Figure 1), and short and thick fingers (Figure 2). The patient had a low and deep voice.

**Figure 3:**
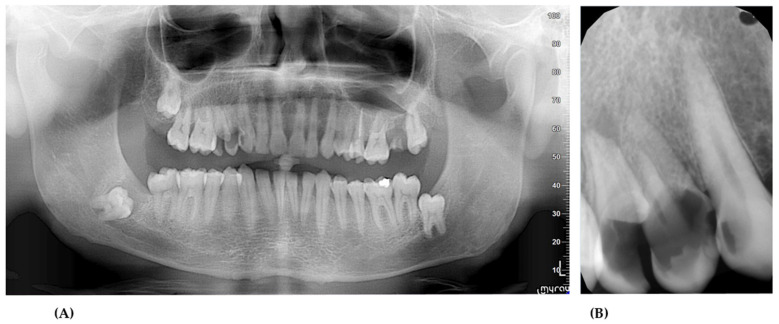
Orthopantomography (**A**) and radiovisiography (**B**) before surgical treatment.

Intraoral examination and additional radiological tests were performed (Figure 3). The patient had periodontitis and teeth 14, 15, and 27 were qualified for extraction. The teeth 13, 12, 25, and 36 were treated conservatively. The patient signed an informed written consent to the procedures and to the use of the data and photos for publication.

Due to the risk of MRONJ, the procedures were performed one at the time and in a minimally invasive manner [10]. An antibiotic prophylaxis was used. The patient started taking the antibiotic one day before the extraction (300 mg of clindamycin three times a day). The procedure was performed using an adrenaline-free local anesthetic (1.8 mL 3% mepivacaine, i.e., 54 mg mepivacaine). In the peri-operative period, mouthwash with 0.2% chlorhexidine solution was recommended twice a day for 10 days. Tooth 14 was removed in a minimally invasive manner. The wound was sutured tightly and without tension. Absorbed polyglycol sutures 4-0 were used. Hemostasis was achieved. A gauze pad was put on and the patient was instructed to bite on it and hold it for 20 min. Antibiotic therapy was continued for the next 7 days (dose of clindamycin, 300 mg, three times a day).

After 48 h, a follow-up visit took place. Wound healing was normal, without pain. The sutures were removed on day 7. Teeth 15 and 27 were removed according to the same protocol (each tooth removed at another visit). The patient had control visits after 7 days, then after 14 days, 6 weeks, 3 months, and 7 months after each procedure. The process of wounds healing was uneventful. Alveolus was filled gradually with normal granulation tissue, resulting from a remodeling hematoma. Finally, the alveolus became covered with normal gingiva.

**Figure 4:**
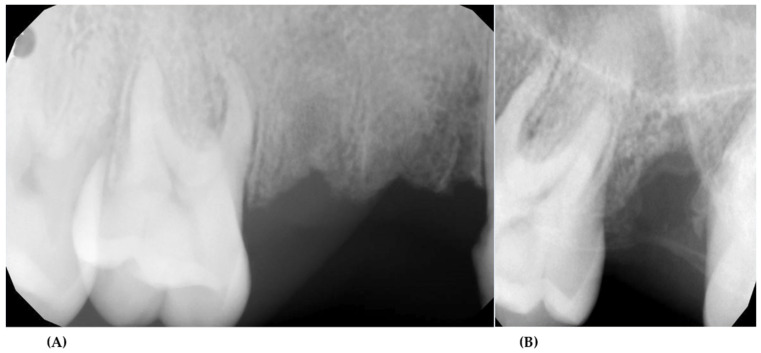
Dental X-rays—7th months after extraction: side after 14 and 15 teeth extraction—normal bone healing (**A**); side after 27 extraction—abnormal bone healing (**B**).

Seven months after the last extraction, the patient came for a follow-up visit. The patient did not report any problems in the oral cavity. The intraoral examination showed complete healing of the 14, 15, and 27 alveoli. Alveoli were fully covered by gingiva. A concave profile of the alveolar ridge was observed in the place of the removed teeth on the right side, which could indicate a vertical loss of bone tissue. Dental X-rays of the 14, 15, and 27 alveoli and photographic images were taken (Figure 4). Radiographic examination revealed a good bone healing at the site of surgery (extraction of teeth 14 and 15). X-ray of the area of tooth 27 showed bone resorption in the distal part of the alveolus. The mesial part showed a correct regeneration.

**Figure 5:**
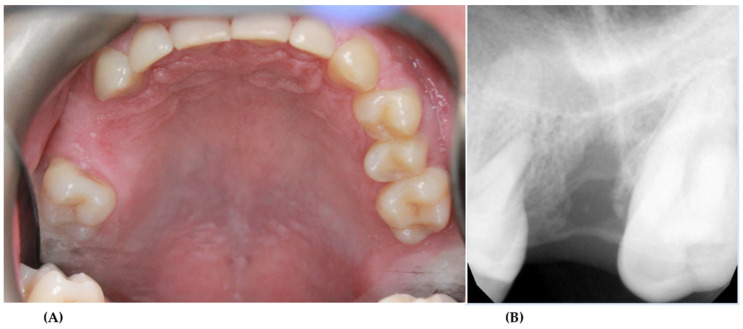
Intraoral photographs after 10th mouths after surgical treatment (**A**); dental X-ray—10th months after 27 extraction—stabilization of bone healing (**B**).

After the next 3 months, the patient reported for the next control. Radiological and clinical examination found healing stabilization in the region of 27 (Figure 5).

## 3. Discussion

Hajdu-Cheney syndrome is a genetic disease and is related to the mutation of the *NOTCH2* gene on chromosome 1 (locus 1p13-p11). The structure of the transmembrane NOTCH receptor is complex and consists of three main parts: an intracellular domain (the so-called PEST domain = polypeptide sequence rich in proline (P), glutamic acid (E), serine (S), and threonine (T)), an intermembrane domain, and an extracellular domain. The receptor is active when it is linked to one of the ligands (JAG1, JAG2, and DLL 1, 2, and 4). In the case of HCS, the mutation concerns a truncation in exon 34 of *NOTCH2*. This results in the loss of the PEST domain and increases in NOTCH2 signaling pathway activity. The mutation in this gene causes a disturbance in bone growth and remodeling, induces an increase in the osteoblast-osteoclast index, loss of bone mass, osteoporosis, and acroosteolysis [10]. In the case of HCS patients, attempts are made to use antiresorptive or antiangiogenic drugs. These drugs aim to slow down progressive osteoporosis and acroosteolysis. Some authors showed an effect on bone density in patients with HCS after the use of bisphosphonates [11]. However, other studies have not shown this relationship [13,14]. In the case of denosumab, an improvement in density was shown, but with no effect on acroosteolysis [18]. In patients receiving BPs or denosumab, a delayed wound healing in the oral cavity and osteonecrosis may occur. Dental procedures involving the alveolar bone process (tooth extractions and bone alveoloplasty), periodontitis, peri-implantitis, sharp edges of teeth, fillings, and prosthetic restorations may be risk factors for MRONJ. Spontaneous osteonecrosis is rarely observed. Other risk factors of MRONJ include the route of antiresroptive/antiangiogenic drug administration, time of treatments, cumulative dosage, additional drugs (e.g., chemotherapeutic agents, glucocorticosteroids), underlying disease (e.g., cancers, osteoporosis), comorbidity (e.g., diabetes mellitus, rheumatoid arthritis), and lifestyle (e.g., tobacco smoking) [31]. MRONJ consists in the destruction of exposed bone, with the exposure persisting for a minimum of 6–8 weeks. Radiographic signs of necrosis are also scored as an early stage of MRONJ [36,37,38,39]. To reduce the risk of MRONJ, the following procedures should be performed prior to the BPs or denosumab treatment: elimination of infectious foci from oral cavity and correction of fillings and removable prosthetic restorations. During BPs or denosumab therapy, patients should be aware of the risk of osteonecrosis of the jaws and the principles of good oral hygiene, and report for follow-up visits every 3–4 months. During visits, hygiene and routine radiological monitoring should be carried out. If surgical treatment is necessary (extractions, resection of root apices, periodontal surgery), it should be minimally invasive. Surgical procedures should be performed in a short-term antibiotic therapy in patients treated with bisphosphonates, denosumab, or bevacizumab. The patient should start taking the antibiotic the day before the surgery and continue the therapy for three consecutive days. Long-term antibiotic therapy (up to 14 days) is used in patients treated with zoledronic acid, taking bisphosphonates intravenously for at least 3 years, or with previous bone inflammation or necrosis. The drugs of first choice are amoxicillin/clavulanic acid for adults at a dose of 1000 mg (875 mg + 125 mg) every 12 h, and in children (45 mg + 6.4 mg)/kg/day in 2 divided doses. If the patient is allergic to penicillin, clindamycin is used in a dose of 300 mg every 8 h in adults, and in children 8–16 mg/kg/day in 3–4 divided doses [36,37,38,39,40,41,42]. According to the recommendations of Campisi et al. [31], a treatment with metronidazole in a dose of 500 mg three times a day should be added the day before the procedure and should be continued for 10 days. Extractions should be performed one at a time and the wound should be sutured [31]. A new idea in the prevention of MRONJ may be the application of platelet concentrates (APCs). APCs can be effective in regulating healing processes and in angiogenesis triggering. Previous studies have not unequivocally demonstrated the effectiveness of APCs in the prevention and treatment of MRONJ. The patient sample size in the studies is small, so further, randomized controlled trials in this area are necessary [36]. The process of tissue healing has to be monitored after surgical procedures. Only if the healing after the performed procedure is uneventful may the extraction therapy be continued. After the procedure, the patient should rinse the oral cavity with a solution of 0.2% chlorhexidine twice a day for 10 days [19,20,21,22,23,31,37,38,39,40,41,42]. Patients should be periodically monitored both clinically and radiologically [36]. If MRONJ occurs and surgery is required, it must include antibiotic therapy (with amoxicillin and metronidazole) and necrotic tissue removal [31]. Alternative therapies may include the following: teriparatide (20 µg *s.c.* per 4 months), pentoxifylline (400 mg *p.o.* twice a day), calcium (1000 mg *p.o.* once a day), vitamin D (min. 2000 UI *p.o.* once a day), vitamin E (400 UI *p.o.* twice a day), ozone (two times a week per 3 min—4 cycles), hyperbaric therapy (40 treatments in hyperbaric conditions are necessary, twice a day for 2 h—100% oxygen, pressure of two atmospheres), biostimulation laser therapy, preparations of platelet-derived growth factors, platelet-rich plasma and plasma rich in growth factors, and recombinant human bone morphogenetic proteins. Alternative therapy is supportive therapy and cannot be used as monotherapy [19,20,21,22,23,31,36,37,38,39,40,41,42,43,44,45,46,47,48,49,50,51,52].

In the case of our patient, we followed the recommendations. Short-term antibiotic therapy was commenced and the patient was monitored clinically and radiographically. The final result of bone healing after the extractions was satisfactory.

## 4. Conclusions

HCS is characterized with the connective tissue disorders, severe bone resorption, and osteoporosis. This is a challenge for professionals who perform oral surgery procedures in patients with HCS. Any interference or injury may result in abnormal healing and even bone necrosis. An additional risk factor is taking antiresorptive or antiangiogenic drugs. This is the first article about an HCS patient treated with denosumab who underwent invasive oral surgery procedures. This case report highlights the difficulties for professionals occurring during the surgery procedures. Management of such patients should be planned and always performed with antibiotic prophylaxis. Periodic clinical and radiological check-ups are very important. In addition to visits to the dentist, the patient should be regularly monitored by the attending physician.

## Figures and Tables

**Table 1:** Brennan and Pauli criteria of Hajdu-Cheney Syndrome.

No	Manifestations	Adults	Children
1	Acroosteolysis	Acroosteolysis and three additional manifestations from 2 to 9 or Acroosteolysis and documented positive family history or Documented positive family history and two additional manifestations from 2 to 9	Any four manifestations from 1 to 9 or Documented positive family history and any other two manifestations
2	Wormian bones or open sutures		
3	Platybasia		
4	Premature loss of teeth		
5	Micrognathia		
6	Coarse face		
7	Coarse hair		
8	Midfacial flattening		
9	Short stature (<5th percentile)		
10	Documented positive family history		

## Data Availability

The data presented in this study are available on request from the corresponding author. The data are not publicly available due to privacy restrictions.

## Abbreviations

APCs	application of platelet concentrates
BPs	bisphosphonates
DLL1	Delta-Like Canonical Ligand 1
DLL2	Delta-Like Canonical Ligand 2
DLL4	Delta-Like Canonical Ligand 4
HCS	Hajdu-Cheney syndrome
JAG1	Jagged Canonical Notch Ligand 1
JAG2	Jagged Canonical Notch Ligand 2
MRONJ	medication-related osteonecrosis of the jaw
*NOTCH2*	*Notch Receptor 2* gene
PEST domain	polypeptide sequence rich in proline (P), glutamic acid (E), serine (S), and threonine (T)
RANK	receptor activator of nuclear factor-κB
RANKL	receptor activator of nuclear factor-κB ligand
